# A Course of Clinical Lectures on Some Important Points of Surgery

**Published:** 1844-11-16

**Authors:** Benjamin C. Brodie


### A COURSE OF CLINICAL LECTURES ON SOME IMPORTANT POINTS OF SURGERY,

*Delivered at St. George's Hospital,*

BY SIR BENJAMIN C. BRODIE.

Gentlemen,—I have two or three more words to say on the disease which formed the subject of the last lecture. In one of the preparations I then showed you, there is a deposit of fibrous matter between the cysts composing the tumour, and binding them together, thus confirming what I said of the manner in which these cysts are aggregated into apparently one mass. I have on the table a tumour taken from a breast which I punctured; but the result of the puncturing was, that afterwards this solid fibrous tumour was formed, and projected above the skin. The fibrinous matter grows generally from the inner part of the cyst, but it is found deposited on the outside also. There is also another disease of the breast which you must be careful not to confound with this; it consists of a membranous bag filled with water, and is widely different from that containing serum. They are found not only in the breast, but also in various other parts of the body, and are in fact the true encysted hydatids. When they occur in the testis, they are situated either between the layers of the tunica vaginalis, or else between the tunica vaginalis and the epididymis; if they are in the course of the cord, they are lodged in the cellular membrane which surrounds it. I have published some cases of this kind, and so has Mr. Cæsar Hawkins. The fluid they contain is pure water, and will all evapoiate. I have seen tumours of this kind dissected out, and have also removed them by external applications. It is necessary you should thoroughly understand this disease, otherwise you will confound it with scirrhus, as has frequently been done. It is important because this is a local disease and may be cured by operation. I have given no name to this affection, because I think it is an error of modern times to be continually giving new names to diseases, but if it must have a name, I think it should be called sero cystic tumour. I purpose calling your attention now to a different subject. In my last lecture, I said I should not always lecture on diseases, but sometimes merely on the symptoms of the disease, because I think in this way a great deal of practical knowledge may be learned. It may be all very well to take up one disease in particular and confine yourself to it; but depend upon this, none will succeed in practice who have not made themselves so far masters of the symptoms, as to be able to judge of the circumstances which produce any one symptom in different diseases. Now there is nothing in practice which you will be so often consulted about, as "PAIN IN THE LOINS." It may be produced by a variety of causes; most commonly it is what is called lumbago, a kind of rheumatic or gouty pain in the lower part of the back and loins. It is a disease which is produced by a general derangement of the system, perfectly independent of any local mischief. You find a patient complaining of severe pain in his loins, aggravated whenever he attempts to move, so that he cannot even turn in his bed. If it is simple lumbago, you will find there has been some derangement of the digestive organs, attended with costiveness; an overloaded state of the colon is sufficient to produce this disease. A gentleman consulted me about a severe pain in his loins, which went off when he got up and bustled about: this was nothing but hardened fæces in the colon, and was entirely removed by an active purgative. Sometimes, however, it is more deeply seated; the patient has acidity of stomach after taking his meals; and is troubled with flatulency; the urine also deposits a precipitate of lithate of ammonia; sometimes it stains the chamberpot like a pink saucer; at other times, it is of a yellow or orange colour; the urine comes away clear, and deposits these sediments most abundantly in the morning. When there are none of these deposits, the urine is clear and healthy. I said this disease was rheumatic, but, in truth, it is gout rather than rheumatism, for all those diseases which occur from the acidity of the stomach approach nearer to gout than rheumatism. The real seat of the disease is in the nerves rather than the muscles; and I say it dwells in the nerves for this reason; that it frequently terminates in sciatica, producing pain throughout the whole extent of the sciatic nerve. That there is inflammation of the neurilemma of this nerve in these affections is proved by several preparations in the Hunterian collection, and in these preparations it will be observed that the neurilemma is much thickened. You may distinguish this affection, then, by its being preceded by costiveness, flatulence, and deposit in the urine. The patient may be much relieved by cupping but this will not be sufficient for his cure; he should be freely purged in the first instance, and this should be repeated frequently. The acidity of the stomach is to be relieved by the administration of alkaline remedies, the best way of administering which is by combining them with a small quantity of some vegetable acid. Sir G. Blaine recommended them to be given in this way. A scruple of carbonate of potash may be taken in the manner I have described, two or three hours after breakfast, and again after dinner. This state of system, if not speedily relieved, lays the foundation for a variety of diseases. In a large number of the cases of synovial inflammation you will find the stomach in this state, and you will in vain attempt the cure by local remedies; the constitutional symptoms are those principally to be attended to. You will also obtain great benefit from the exhibition of small doses of colchicum, from i. to ij. grs. of the acetous extract, at bed time, for several successive nights. I am not in the habit of giving this medicine in large doses. There is another form of the disease which is very similar to this, in which there is intense pain, extending down the limb, and attended with a good deal of fever; by and by the limb becomes numb, and the muscles do not obey volition; then the pain in the back subsides, and the patient is left in a state of paraplegia. This is not a very common disease, still you may meet with it; it is similar to lumbago but more intense. If the disease is allowed to go on, the paraplegia is hopeless: the symptoms resemble those of inflammation of the spinal cord; but as patients do not die of this malady, it was very long before I had an opportunity of confirming this opinion. I was called to see a gentleman who was suffering from severe pain in the loins, accompanied by incipient paraplegia of the lower limbs. I prescribed a course of mercury but it had no effect, and he went into the country again, not at all relieved. About twelve months after I saw him again; he was in the same unfortunate condition. Like the King in the Arabian nights, his lower limbs were like marble; it so happened that when I called there, I was on my way to Dr. Davies, to see some preparations, and there I saw the spinal cord and corda equina encrusted with coagulated lymph; he described the symptoms of the patient from whom it was taken, and they entirely corresponded with those of my own patient, only that his patient died, whereas mine recovered. I suppose if allowed to run its course, many would die; still more would remain paraplegic. But I believe much may be done for this distressing affection. If there is inflammation of the cord, treat it as you would inflammation of the membrane of the cord; purge the patient, cup him in the loins, and bring him as soon as possible under the influence of calomel and opium. I am sure I have saved many by this treatment. Again, pain in the loins may be the consequence of diseased kidneys; this is sometimes a nice point to decide, as you cannot judge from the pain. The kidney may be much diseased without giving much pain, or the patient may suffer the most exquisite pain if there be a calculous; but in this case it will only be felt whilst it remains in the ureter. No pain varies so much as that produced by calculi, but generally it is aggravated by any excess. Sometimes, the patient has great weakness in the loins; the pain arising from such weakness, being relieved by placing a cushion under him when he lies down; but the nature of the pain is not sufficient to enable you to say whether or not it originates in the kidneys.

Swelling and inflammation of the testicle, accompanying severe pain in the loins, is generally indicative of disease of the kidneys. If there is cutting pain on making water, attended with sickness, the same disease is to be suspected, as nearly all the diseases of the kidneys are marked by the irritable state of the stomach. When this is the case the patient is languid, incapable of exertion, his appetite is bad, he loses flesh; but if these symptoms are not sufficient to satisfy you as to the nature of the disease, you must examine very carefully the state of the urine. In most cases, where pain in the loins is the result of diseased kidneys, the urine is found to be albuminous. This may be known by boiling a small quantity, when it will become opaque from the deposition of its albumen; or the same thing results from the addition of nitric acid; if it should be voided opaque, it is rendered more so by the means I have stated. Sometimes the urine deposits pus, or something so much like it as not to be distinguished from it; as the disease goes on, other symptoms occur which clearly prove it to be in the kidneys. If there be a calculus formed there, it will some day or other escape into the bladder; but if its passage from the kidneys should be obstructed, masses of lymph are formed, which passing down the ureters, produce the same distressing pain in the loins and testicles as that arising from stone. These masses of lymph sometimes present a laminated appearance, and are of an oval form; at other times they are found mixed with pus. Occasionally there is a large secretion of mucus in the kidneys, which, in its passage through the ureters, produces very severe pain. But you are not to conclude that a patient has diseased kidneys, merely because he has pain in the loins, as there are many other symptoms to be considered. Caries of the vertebræ is another cause producing pain in the loins. When the vertebræ are affected with scrofulous disease, the cancellous structure is infiltrated with cheesy matter. Ulceration of their cartilages is also found in children of strumous habit. There is another disease attended with the same symptom: it appears to be a kind of rheumatic inflammation of these bones, or cartilages, or it may be of both. It occurs chiefly in adults, and is in no way connected with a scrofulous diathesis. Either of these causes, then, may produce pain in the loins; the difficulty is to determine from what cause it arises in any particular instance. In scrofulous disease, pain is generally very trifling, sometimes so slight as to be scarcely observable; perhaps, when the patient is walking, he strikes his foot against a stone, which jars his back and produces slight uneasiness; he also feels it on stooping, or a slight blow may give rise to it. But, in many cases, the symptoms are so slight that you are not justified in setting it down as diseased vertebræ till the appearance of lumbar abscess, which is a result of disease of the bone, and not of the soft parts.

Mr. Abernethy was unquestionably wrong in his opinions on the origin of this disease. I do not recollect ever to have examined a case where the bone was not diseased. In the other form of disease which I mentioned, the pain in the loins is considerable, and is aggravated by motion; but still the pain is very different from lumbago, coming on gradually, whereas the latter, as you are aware, comes on in paroxysms. Again, in lumbago the pain extends to the hips and thighs, but this is confined to the back. If allowed to go on, its nature becomes plain enough at last. Here the bodies of the vertebræ are affected; the consequence of which is a tilting up of their spinous processes, ending at last in lumbar abscess.—This elevation of the spinous processes is what is called angular curvature. Lumbar abscess may then be the result of inflammation of the vertebræ as well as of scrofulous disease; the distinction being that in the former cases the matter does not escape externally, but in the latter it does. It is quite an extraordinary thing, the extent to which caries may take place in bones, without matter being formed. But pain may occur in this region when the disease is in the vertebræ above; in disease of the dorsal vertebræ, for example, the pain is referred to the loins: this is of course sympathetic pain; but when it has gone on for a year or two, it is felt in the actual seat of disease also. I said that when there was pain in the loins, with deposits in the urine. the kidneys were always diseased; but I am not quite satisfied that this sign is sufficient. I attended a gentleman some time ago, with Dr. Prout; we examined his urine, and thought he had mulb rry calculi, and treated him accordingly; his urine was sometimes of a greenish colour. When he struck his foot against the pavement in walking, he suffered slight pain in the back, which led me to suspect some disease of the spine. But the knowledge we then possessed of these diseases was insufficient to determine whether or no such was the case. He went into the country, but I saw him again some time after, and then, on examination, I found very considerable angular curvature of the spine, two or three of the vertebræ being affected; I then treated it as caries of the spine, and he recovered, the urine no longer remaining albuminous. You must have observed in patients brought in here after a fall, how frequently the urine is deranged; it is not then to be wondered at if it should be considerably affected when there is actual disease in the vertebræ. In these latter cases, however, the urine is rendered ammoniacal as well as albuminous. There is still another disease which I must mention as producing pain in the loins; it somewhat resembles fungus hæmatodes; in this disease, it is the lumbar nerves which appear to be affected; it is attended with very severe pain, but is not at all common. You may be led to suspect the presence of this disease, by the absence of those symptoms which I have described as indicative of the other affections of this region: by the pain extending down the whole of the limb; and your diagnosis will be rendered certain by a tumour being felt in the loins, which may be detected by pressing upon the abdomen. Weakly persons are very subject to pain in the loins, independent of any disease of the part; half the delicate women in this town, from their habits of living, suffer from this pain, and you can never ask an hysterical young woman to relate her symptoms, but she will mention pain in the loins. I have seen at least forty cases of this kind. With such persons, you will always find there is exquisite tenderness of the integuments over the part; but this is only so long as the patient's attention is directed to it; avoid this, and you may press it as much as you please. This is to be detected by the cold hands and feet, irregular menstruation, in some cases the existence of some other discharge, and by a sensibility of the skin when the patient's attention is directed to the part. This class of patients are not to be implicitly relied upon; as they will sometimes tell you the pain is slight, at other times severe, but most frequently they endeavor to exaggerate their symptoms; it is, therefore, necessary that you should be very careful in distinguishing between this and other affections. I need not tell you how important it is to distinguish actual disease from nervous debility.—*London Med. Times.*

